# Multi-country willingness to pay study on road-traffic environmental health effects: are people willing and able to provide a number?

**DOI:** 10.1186/1476-069X-13-35

**Published:** 2014-05-09

**Authors:** Tifanny Istamto, Danny Houthuijs, Erik Lebret

**Affiliations:** 1 Institute for Risk Assessment Sciences (IRAS), Utrecht University, Utrecht, The Netherlands; 2 National Institute for Public Health and the Environment (RIVM), Bilthoven, The Netherlands

**Keywords:** Air pollution, Noise, Willingness to pay, Don’t know, Protest vote, Contingent valuation, Open-ended, Survey, Questionnaire, Uncertainty

## Abstract

**Background:**

The health impacts from traffic-related pollutants bring costs to society, which are often not reflected in market prices for transportation. We set out to simultaneously assess the willingness-to-pay (WTP) for traffic-related air pollution and noise effect on health, using a single measurement instrument and approach. We investigated the proportion and determinants of “protest vote/PV responses (people who were against valuing their health in terms of money)” and “don’t know”/DK answers, and explored the effect of DK on the WTP distributions.

**Methods:**

Within the framework of the EU-funded project INTARESE, we asked over 5,200 respondents in five European countries to state their WTP to avoid health effects from road traffic-related air pollution and noise in an open-ended web-based questionnaire. Determinants of PV and DK were studied by logistic regression using variables concerning socio-demographics, income, health and environmental concern, and risk perception.

**Results:**

About 10% of the respondents indicated a PV response and between 47-56% of respondents gave DK responses. About one-third of PV respondents thought that costs should be included in transportation prices, i.e. the polluter should pay. Logistic regression analyses showed associations of PV and DK with several factors. In addition to social-demographic, economic and health factors known to affect WTP, environmental concern, awareness of health effects, respondent’s ability to relax in polluted places, and their view on the government’s role to reduce pollution and on policy to improve wellbeing, also affected the PV and DK response. An exploratory weighting and imputation exercise did not show substantial effects of DK on the WTP distribution.

**Conclusions:**

With a proportion of about 50%, DK answers may be a more relevant issue affecting WTP than PV’s. The likelihood to give PV and DK response were influenced by socio-demographic, economic and health factors, as well as environmental concerns and appreciation of environmental conditions and policies. In contested policy issues where actual policy may be based on WTP studies, PV and DK answers may indeed affect the outcome of the WTP study. PV and DK answers and their determinants therefore deserve further study in CV studies on environmental health effects.

## Background

Epidemiological studies have extensively documented the health effects of traffic-related air pollution, e.g. an increased risk for heart attacks, exacerbation of asthma in children and a reduction in life expectancy, and noise, e.g. noise annoyance, sleep disturbance, hypertension and cardiovascular effects, and poorer school performance [1-6]. These health and wellbeing impacts induce costs to society, which are, to a large extent, external costs as they are not reflected in the market price for transportation and are not taken into account in the allocation of economic resources [7]. It is increasingly recognised by e.g. the European Ministerial Conferences on Environment and Health and WHO that in order to effectively and efficiently manage environmental quality, it is necessary to take into account all costs and benefits of alternative policy scenarios and to develop ways for the internalisation of external costs [8]. The assessment of willingness-to-pay (WTP) is a common approach to estimate external costs.

In our study, we were interested in expressing the health impacts of both traffic-related air pollution and noise in terms of monetary value, as one of the ways to aggregate across the multiplicity of health effects documented in the literature. Screening of the literature revealed that mainly two different approaches and instruments were used to assess the monetary costs of health and wellbeing effects of air pollution and noise. In addition, relatively few studies provide original data on the costs of traffic-related health impacts. In air pollution studies, the expressed or stated WTP-approach using contingent valuation/survey-based economic methods is dominant [9-11]. For noise the revealed WTP through hedonic pricing approaches is commonly used, where differences in property values and the association between noise exposure and real estate prices are used to estimate WTP [12]. As a result of these different approaches, the costs of traffic-related air pollution and of noise are difficult to compare, since potentially different costs elements are included in these different methods. It is, for instance, unclear which specific health and wellbeing effects of noise are driving the relations observed in hedonic pricing methods. Presumably, these are observable effects such as annoyance and sleep disturbance, since most buyers may not be aware of noise effects on blood pressure, heart attacks and cognition in children. Moreover, the association between real estate value and noise may be confounded by traffic-related air pollution and street safety aspects. Similarly, the estimates from stated WTP to avoid effects from air pollution may depend on information provided to respondents, the phrasing of the questions and the context of the study. In short, the values from monetisation studies on air pollution and noise are difficult to compare because different valuation methods are being used. This can hamper integrated health impact assessments of traffic policies [13].

We therefore set out to simultaneously assess the WTP for traffic-related air pollution and noise effect on health, using a single measurement instrument and approach. We did this in the EU-funded research project INTARESE (Integrated Assessment of Health Risks from Environmental Stressors in Europe) [14]. Our general objectives were to assess and compare the monetary value of air pollution and noise effects based on WTP, to compare within and between country differences, and to study determinants of such differences. In this manuscript, we focus on the willingness of respondents to answer WTP questions and on the ability to provide quantitative WTP answers.

### The WTP concept for environmental health impacts

Willingness-to-pay (WTP) is a central concept in the assessment of the external costs of environmental pollution. It attempts to translate people’s preferences for environmental goods, e.g. clean air and a quiet living environment and willingness to avoid environmental health effects. It describes the monetary equivalent of the loss of welfare related to intrinsic values. However, contrary to regular commodities, the economic value of environmental good is not easily expressed based on a market price [15].

There are several components to the potential loss of welfare. One component comprises various costs that illness imposed on a particular individual and society, imposing real (treatment) related expenses. These are generally referred to as “Cost-of-Illnesses (COI)” and can often be derived from market prices of medical consumption. COI can be determined in a narrow sense (taking only direct medical costs) or broader (caregiver’s time, productivity losses, etc.), thus the COI is often considered as a lower-bound of the WTP to avoid environmental health effects [16]. The second component of loss of welfare is the sheer disutility of the illness, e.g. reduced enjoyment of desired leisure activities, stress, pain, suffering, anxiety, or inconvenience to the individuals affected, their family and communities they live and work in. Yet another component of WTP covers the price of the (public) good of a clean environment per se. This may include the WTP for one’s private use of the good, as well as an altruistic component contributing to a clean and safe environment for others.

The WTP can be assessed by directly asking the individuals what the good or the effect is worth to them using the survey-based method of contingent valuation (CV) approach [11]. This approach is the most commonly applied method for valuing preferences for non-market goods with thousands of applications conducted to date [10]. In essence, the CV approach directly or indirectly questions respondents about their WTP through open-ended questionnaires, bidding games, payment card methods or alternatives thereof. The WTP values thus may cover private costs as well as public good costs, depending on context and value system of respondents.

Several studies identified respondents who indicated a “protest vote/PV” or “protest response” [17-20], thus recognising lack of full alignment of the WTP concept with judicial or ethical principles (notably the “Polluter pays principle”). For air pollution, a recent study [21] reported 11% PV. A study on traffic noise [22] reported that nearly half of their respondents reported PV (49%). Studies on ecological values have reported the protest votes, e.g. 21% PV for forest biodiversity [19] and nearly 80% for ensuring the bathing water in some areas in the UK to meet EC standard [23], thus illustrating the importance of the context.

An implicit assumption in WTP studies is that respondents can provide, with full certainty, responses to the valuation question that reflect their true valuation of the good. This is often not the case. Respondents often are unfamiliar with the valuation of external costs of environmental health effect and may have difficulties answering the valuation questions [24-26]. The inability to express WTP may stem from unfamiliarity of the effects involved. This may partly be reduced by providing information about the nature of the effects prior to the WTP question [27,28]. The inability to express WTP may also originate in the inherent difficulty to put a price on external costs in the absence of market prices [29,30]. Even COI can be difficult to estimate in many health care systems where health insurance directly covers the health-related costs. Currently, little is documented about “don’t know/DK” answers in quantitative terms, since DK answers are usually not allowed or not directly assessed and reported. In some cases, uncertainty about WTP value is assessed in follow-up questions by specifying the degree of (un)certainty in the WTP answers. For instance, the NEEDS (New Energy Externalities Developments for Sustainability) study asked: “Are you confident in your WTP answer?” where 35% of the respondents were either not confident in their WTP answer or had “missing” data on this question [21]. The effect of the lack of confidence on the WTP values was not reported, however.

### Research questions regarding protest votes and don’t know answers

To address the issues of PV and DK answers, we formulated the following specific research questions for our study: i) what percentage of respondents is unwilling to express WTP; alternatively, which percentage gives a “protest vote/PV” response?; ii) what percentage of respondents is able to quantitatively express WTP; alternatively, which percentage gives a “don’t know/DK” answer?; iii) which factors are associated with PV and DK answers?; and iv) how is the distribution of WTP responses affected by DK answers? We explored these questions using three sets of vignettes with different levels of information about the health and wellbeing effects of traffic-related air pollution and noise. In addition, we used variables known to affect WTP to shape the framework for PV and DK analysis of determinants. This is elaborately in the Methods section.

## Methods

To assess economic values of health effects for air pollution and noise and with a single instrument and approach, we used a web-based questionnaire survey, carried out in the United Kingdom (UK), Finland (FI), Germany (GE), the Netherlands (NL) and Spain (SP), within the framework of the INTARESE project. We used an open-ended questionnaire to avoid anchoring effects associated with alternative CV approaches. Anchoring or starting point bias is the phenomenon known from the economic and psychological sciences that might affected respondent’s answers by presenting (a range of) values prior to the WTP questions, as may be the case in the payment card method [31]. A high initial value may lead respondents to give higher values than without such a cue. Avoiding anchoring effects was important in our study where we were interested in the valuation of a variety of different health and wellbeing outcomes reported in the literature for traffic-related air pollution and noise. The web-based survey was conducted in December 2010.

### Study population

An external survey agency (Blauw Research, ISO9001 & ISO20252 certified) recruited respondents through their existing population panels in the five countries. This survey agency employs an active panel recruitment process using multi-sourced recruitment methods to contact each layer of the population in nationally representative context. The data collecting covered a period of two weeks. Panellists were invited to participate through the regular panel procedures (e-mail) and received a personal login code and password to fill in the web-based questionnaire. Progress was monitored on a daily basis. Quality assurance included online questionnaire check, daily helpdesk mail check with immediate follow-up, sending out a reminder, check on interview duration. By weighting on age, sex and education, the sampling was representative for the population of the specified countries, aged 18 to 64 years old. We aimed and obtained 2,000 respondents per country. Non-respondents were replaced by alternative panel members with similar age, sex and education. No follow-up non-response information was collected.

### The questionnaire

As described earlier, the questionnaire consisted of the three main groups of questions obtained from the literature in different domains. These are: i) social-economic factors, recognised in the economics literature and others, i.e. household income, gender, education/socio-economic status, ii) factors known from the public health field, i.e. severity of health effects, familiarity with the effects, current health status, and iii) factors from the social sciences domain, i.e. familiarity and perception of risks, level of awareness, level of concern of environmental health effects, perceived level of exposure. We adopted, where possible, widely used and standardised questions and scales. Two versions of the questionnaire were made to limit size and cognitive burden to the respondents; one focusing on road traffic-related air pollution and the other on road traffic noise. Respondents randomly received questions on air pollution or noise to prevent biases. The questions on the WTP in general were followed by a series of questions on the health gain in specific effects of air pollution or noise related to a reduction in pollution level.

The questionnaire started with several questions on how respondents perceived their health, based on the standardised and validated Health Survey RAND-36 [32] for general health score. In this part, respondent’s concern regarding the specific pollutant was also inquired. In the second part of the questionnaire, respondents were provided with a brief description of the health effects related to road traffic air pollution and noise; we only presented those health effects for which authoritative reviews indicate sufficient scientific evidence. The third part of the questionnaire assessed social-demographic information such as age, gender, education, household net income per month and how respondents perceived the level of environmental concern in general.

Three vignettes were used for the brief descriptions in the second part of the questionnaire. The first was a generic qualitative description of the health effects for which there is sufficient evidence in the literature. This addresses the “naïve” understanding of respondents of the health effects. For air pollution, these were risk for hospital admission for cardiovascular- and respiratory diseases, reduction of life expectancy, and risk for doctor-diagnosed asthma in young children. For noise effects, these were risks for heart attacks, severe sleep disturbance, severe annoyance, and poorer reading performance in children. We also assessed whether respondents were aware of these health effects. The second vignette was a quantitative description of a single specific health effects, in line with other recent WTP studies on air pollution and noise. This addresses the current scientific practice of contingent valuation studies. For air pollutions, it explained that a 50% decrease in the air pollution emissions by 2030 was related to half a year gain in average life expectancy. For noise, an increase from 50 dB to 65 dB meant an increase of 13% to become severely annoyed by noise. The third vignette was a quantitative description of a scenario of combined effects that would happen simultaneously if a certain policy would be implemented (a more policy-oriented approach). We presented a set of quantitative changes in risk for the health effects described in the general description. The wording of these three vignettes is provided in Additional file 1.

Respondents were provided with the option “I don’t know” as an answer to the questions about WTP amounts to avoid them giving an irrelevant answer or for the sake of going to the next questions. If respondents answered €0 on the WTP general questions, follow-up questions were asked about their motive for the zero response. Options for these follow-up questions were: i) costs should be included in transportation prices; ii) government should pay all costs to reduce air pollution; iii) effects of air pollution from road traffic are negligible; iv) principally against putting amount of money on health; and v) other reason. Options i), ii), or iv) of these follow-up questions, combined with the WTP of €0, were used to identify a protest vote (PV), an answer indicating that respondents did not accept the concept of WTP.

The questionnaire was first pre-tested on length and comprehensibility by colleagues and by professionals from the survey bureau. Then, the questionnaire was translated into the languages of participating countries. Subsequently, the translations were checked by native speakers on translation and comprehensibility (project members of the INTARESE project). Finally, the questionnaire was pre-tested in 10% of the samples in the main study. At the end of the questionnaire, respondents could provide their feedback. The pre-test indicated that a) many respondents volunteered that this was an important topic to address, and b) many respondents indicated that the WTP questions were difficult to answer. This strengthened our view that respondents should have an option to give a “don’t know” answer to the WTP questions.

### Payment vehicle

The payment vehicle describes the manner in which the payment of the WTP amount is made and specifies the timing of the payment. This study applied an out-of-pocket voluntary payment vehicle. We asked respondents their annual contribution for the rest of their lives. For example: “What is the maximum amount of money you personally are willing to pay annually for the rest of your life to avoid 100 additional cases per 10,000 children of poorer reading performance due to traffic noise?”. We reminded respondents to take their household income into account prior to answering the WTP question.

### Statistical analysis

After data cleaning, recoding and explorative descriptive analyses, we applied a 1.5% cut-off point for WTP values as default, to avoid unrealistically high values for WTP. This cut-off is similar to values reported in the literature; this roughly corresponds to a cut-off based on expendable income of €3000 per person/month [21]. Thus, all reported means and medians are trimmed. Where necessary, we converted the values of national currencies into Euro’s. WTP values are presented in € per person per year (€ pp/y).

To assess which factors affected the PV and DK responses, we used multiple logistic regression models for the binomial distributions of PV and DK. In lieu of a clear framework in the literature for determinants of PV and DK answers, we explored the role of independent variables identified and reported in the literature on WTP (e.g. age, gender, years of education, household income, financial position, general health score, country), together with factors such as environmental concern, awareness about the increased health risks associated with road traffic-related air pollution or noise, severe concern about the health effects of air pollution or noise, sensitivity to road traffic air pollution/noise, difficulty to relax in a place with air pollution/noise, the confidence in the government to reduce road traffic air pollution/noise, and the respondent’s opinion on policy attempting to reduce road traffic air pollution/noise with the aim to improve the wellbeing of residents. The 5-point scale for perception related variables was converted into a smaller “agree, neutral or disagree”-scale or “yes or no”-scale. The 10-point scale to indicate the level of concern was dichotomised into “very concerned” or “not very concerned”, with the score of 8–10 for “very concerned”. The 3-point scale of awareness was converted into a binomial “yes or no”-scale where those who chose the option of “very much aware” were categorised into a “yes” answer on a binominal scale.

Since the DK responses were more prevalent than the PV, we only explored the possible influence of the DK responses on the WTP distributions. We applied two approaches: weighting and imputation, both based on the factors in the multiple logistic regression models. Weighting is the standard method of non-response adjustment for surveys subject to unit response and is a natural extension of weighting for sample selection where respondents and non-respondents are classified into adjustment cells based on covariate information for both group [33]. It is also applied for item non-response [34]. For the weighting, we obtained from the logistic regression model the probability of response. The inverse of the predicted probability from this model was then used to adjust the sampling weight. The sampling weight per country for each respondent was then multiplied by the non-response weight to obtain a combined weight for subsequent analysis. By weighting per country, we were then able to find pooled estimates of WTP.

For the imputation, we applied a two-step approach, given the distribution of the known WTP’s, the large fraction of zero’s and a skewed continuous distribution,. Multiple-imputation uses the distribution of the observed data, in our case the WTP values and the factors used in the logistic models, to estimate plausible values for the unknown WTP’s. First, unknown WTP values were imputed as “zeros” and “ones” with a logistic model. Subsequently, respondents with an imputed WTP values of “one” were assigned a new imputed WTP values based on a (truncated) linear regression model where WTP values were log-transformed with a minimum value of €1 and a maximum upper boundary that was equal to the criteria of a 1.5% cut off value. The imputation was repeated 50 times.

Since the percentages were rounded in this study, summing percentages (%) may not add up to 100%. All of our analyses were performed with IBM Statistics SPSS Version 19, with the exception of the imputation that was carried out with Stata 13.

## Results

We had 10,464 respondents participating in our study, of which 5,243 filled out the air pollution part and 5,251 the noise part. The respondents in our study were representative for the population of the specified countries based on age, gender and education. Average household net income of our respondents compared well with statistics from European Statistics/Eurostat [35]. The characteristics of the participants are described in Table 1.

**Table 1:** Social-demographic, health- and perception related factors in our sample

		**Air pollution**	**Noise**
		**In percentage (%)**	
Respondents per country	NL	19.8	18.7
	UK	19.4	19.5
	DE	20.8	21.0
	ES	20.2	20.0
	FI	19.8	20.8
Age group	18 - 24	13.6	13.5
	25 - 34	21.6	20.8
	35 - 44	25.0	23.8
	45 - 54	22.3	22.9
	55 - 64	17.4	18.9
Gender	Female	51.7	52.8
Environmental concern in general	Low	25.3	26.4
	Medium	36.4	35.3
	High	38.3	38.3
Financial position (have you experienced difficulties in the last 12 months to live on your household income?)	No, not a problem at all	24.5	25.6
	No, not a problem, but have to be careful with expenditures	35.6	34.9
	Yes, with a slight difficulty	27.1	27.7
	Yes, with a large difficulty	12.9	11.8
Household net income per month	€1000 or less	22.2	22.8
	€1001 to €1500	19.4	19.5
	€1501 to €2000	17.9	17.7
	€2001 to €3000	21.2	20.1
	> €3001	19.3	19.8
Awareness about the increased health risks due to air pollution/noise	Yes	66.2	33.0
Severe annoyance by air pollution/noise	Yes	12.9	11.4
Severe concern about the health effects of air pollution/ noise	Yes	22.6	19.9
Constant freight traffic nearby dwelling	Yes	7.9	7.7
Sensitive to road traffic air pollution/noise	Yes	32.5	40.7
Difficulty to relax in a place with air pollution/noise	Yes	55.5	68.0
Confidence in the government to reduce road traffic air pollution/noise	Agree	25.7	22.8
	Neutral	30.9	33.3
	Disagree	43.3	43.8
Policy on road traffic air pollution/noise is not aimed at improving the wellbeing of residents	Agree	46.1	41.0
	Neutral	36.8	39.0
	Disagree	17.1	20.0
		**Average of all 5 countries**	
Average years of education		14.3	14.1
Average general health score (0 = worst; 100 = best)		62.0	61.6

The general personal characteristics in Table 1 were comparable for the air pollution and the noise respondents, but the pollutants were perceived differently. The awareness of the health risk associated with air pollution was twice as high as for noise and the sensitivity for the pollution and the difficulty to relax in a polluted place, was higher for noise than for air pollution.

### Protest votes/PV and don’t know/DK

Table 2 presents the proportion of respondents with PV and DK answers for the three vignettes of WTP questions, with clearly higher proportions for DK than PV responses. Approximately 10% of responses were PV for WTP. The main reasons to give a zero WTP value to reduce air pollution were: (i) costs should be included in transportation prices (30%), (ii) government should pay all costs to reduce air pollution (30%), (iii) principally against putting amount of money on health (20%), (iv) effects of air pollution from road traffic are negligible (5%) and (v) other reason (15%). The first three categories constituted the PV’s. For noise, the percentages were similar: (i) 26%, (ii) 33%, (iii) 20%, (iv) 7%, and (v) 14%. Table 2 indicates that a large proportion of respondents did not know how to answer WTP questions, also when more quantitative information was provided. The percentage of DK answers was 4-8% higher for the specific health effect and scenario-based WTP questions, compared to the general WTP questions.

**Table 2:** The proportion of PV (protest vote responses) and DK (“don’t know”- responses)

	**Air pollution: % of respondents**	**Noise: % of respondents**
PV	8.6	10.9
DK for general WTP	48.0	47.4
DK for specific effects	55.7	53.8
DK for scenario of combined effects	53.8	51.4

Respondents were asked how much they were willing to pay in general, for specific air pollution or noise effects, and for a scenario of combined effects.

### Variables associated with PV for air pollution and noise

The results of the logistic regression analysis for PV are presented in Additional file 2: Table S1. Air pollution PV was significantly associated with age, gender, education, country (with respondents from UK and Finland having a lower probability for PV), large financial difficulties, environmental concern, difficulty to relax in polluted places, and disagreement with the statement that the government was doing their best to reduce air pollution. Noise PV was significantly associated with age, country (Finland with lower probability for PV), the general health score, environmental concern, disagreement with the statement that the government was doing their best to reduce noise, a neutral opinion that policy on air noise was aimed to improve wellbeing, and severe freight traffic nearby dwelling. Common determinants, although not always in all of the categories, for PV air pollution and noise were: age, gender, country, financial position, environmental concern, and the opinion on government’s attempt to reduce pollutants.

### Variables associated with DK for air pollution

The factors associated with the DK for air pollution in the multiple logistic regression analysis are shown in Table 3. The OR’s for these variables were generally the same for the three types of WTP questions, although significance levels varied as indicated by 95% of confidence interval. Several variables had a significant influence on the probability of DK answers for all three vignettes, e.g. social-demographic variables such as age and gender. In addition, the environmental concern and the respondent’s view on the government’s role to reduce pollutants and on their policy to improve wellbeing were also associated with DK response. The German respondents were the least likely to provide a DK answer. The Finnish respondents had the highest likelihood to give a DK response to the generic WTP question. FP had a significant effect on the probability to provide WTP for air pollution general effects (OR’s were also below 1 for the other two vignettes, although they did not reach statistical significance). In other words, the more financial difficulties respondents experienced, the more they were able to provide general WTP. Severe air pollution concern and the ability to relax in a place with air pollution were significant with the DK for scenario. In addition, awareness of health effects associated with air were significant for specific effect and the scenario, but not for the general effect. The degree of annoyance due to air pollution and the severe freight traffic nearby the dwelling did not affect the DK. Respondents who had answered neutral to policy related-questions were more likely to provide a DK response.

**Table 3:** Odd ratios and confidence interval of variables associated with DK for air pollution in multiple logistic regression analysis

	**Air pollution**					
	**DK WTP general**		**DK WTP life expectancy**		**DK WTP scenario**	
	**O.R.**	**95% C.I. for O.R.**	**O.R.**	**95% C.I. for O.R.**	**O.R.**	**95% C.I. for O.R.**
Age group						
18-24 (baseline)	1	[0 - 0]	1	[0 - 0]	1	[0 - 0]
25-34 (1)	1.32	[1.07 - 1.62]	1.28	[0.96 - 1.72]	1.62	[1.32 - 1.98]
35-44 (2)	2.05	[1.67 - 2.52]	2.09	[1.56 - 2.81]	2.20	[1.79 - 2.70]
45-54 (3)	2.08	[1.68 - 2.57]	1.82	[1.34 - 2.46]	2.13	[1.72 - 2.63]
55-64 (4)	2.32	[1.85 - 2.89]	2.46	[1.79 - 3.38]	2.30	[1.84 - 2.87]
Gender female (1)	1.76	[1.56 - 1.99]	1.77	[1.49 - 2.10]	1.79	[1.59 - 2.03]
Education per 10 years	0.89	[0.79 - 1.00]	0.86	[0.72 - 1.01]	0.82	[0.73 - 0.92]
Country NL (baseline)	1	[0 - 0]	1	[0 - 0]	1	[0 - 0]
Country UK (1)	1.01	[0.83 - 1.23]	0.80	[0.60 - 1.06]	0.97	[0.79 - 1.18]
Country DE (2)	0.75	[0.62 - 0.92]	0.62	[0.47 - 0.82]	0.70	[0.57 - 0.85]
Country ES (3)	1.04	[0.84 - 1.29]	1.04	[0.76 - 1.41]	1.00	[0.80 - 1.24]
Country FI (4)	1.34	[1.11 - 1.63]	1.08	[0.81 - 1.42]	0.98	[0.81 - 1.19]
Financial position - not a problem at all (baseline)	1	[0 - 0]	1	[0 - 0]	1	[0 - 0]
FP – not a problem, but have to be careful (1)	0.82	[0.70 - 0.96]	0.97	[0.77 - 1.21]	0.95	[0.81 - 1.11]
FP – with a slight difficulty (2)	0.83	[0.70 - 0.99]	0.84	[0.65 - 1.08]	0.88	[0.74 - 1.05]
FP – with a large difficulty (3)	0.75	[0.60 - 0.94]	0.78	[0.57 - 1.08]	0.91	[0.72 - 1.14]
Household net income: €1000 or less (baseline)	1	[0 - 0]	1	[0 - 0]	1	[0 - 0]
Hh income €1001 to €1500 (1)	0.85	[0.70 - 1.02]	0.87	[0.66 - 1.13]	0.76	[0.63 - 0.92]
Hh income €1501 to €2500 (2)	0.65	[0.53 - 0.79]	0.64	[0.48 - 0.85]	0.64	[0.52 - 0.78]
Hh income €2001 to €3000 (3)	0.64	[0.53 - 0.78]	0.56	[0.43 - 0.73]	0.61	[0.51 - 0.74]
Hh income > €3001 (4)	0.64	[0.53 - 0.78]	0.61	[0.46 - 0.81]	0.61	[0.50 - 0.75]
General health score per 25	1.29	[1.14 - 1.46]	1.06	[0.89 - 1.26]	1.24	[1.09 - 1.40]
Awareness of health effects associated with air pollution - aware	0.91	[0.80 - 1.04]	0.82	[0.68 - 0.99]	0.87	[0.76 - 0.99]
Environmental concern: Low (baseline)	1	[0 - 0]	1	[0 - 0]	1	[0 - 0]
Environmental concern: Medium (1)	1.33	[1.13 - 1.56]	1.39	[1.10 - 1.74]	1.19	[1.01 - 1.39]
Environmental concern: High (2)	1.14	[0.96 - 1.35]	1.34	[1.05 - 1.72]	1.00	[0.84 - 1.19]
Severe air pollution concerns – yes (1)	1.12	[0.94 - 1.34]	1.22	[0.94 - 1.58]	1.23	[1.03 - 1.47]
Sensitive to air pollution – yes	0.91	[0.78 - 1.06]	0.94	[0.76 - 1.18]	0.89	[0.77 - 1.04]
Difficulty to relax in a place with air pollution - yes	0.85	[0.74 - 0.97]	0.94	[0.77 - 1.14]	0.86	[0.75 - 0.98]
Government doing their best to reduce air pollution - agree (baseline)	1	[0 - 0]	1	[0 - 0]	1	[0 - 0]
Government doing their best to reduce air pollution - neutral (1)	1.28	[1.09 - 1.50]	1.51	[1.19 - 1.91]	1.22	[1.03 - 1.44]
Government doing their best to reduce air pollution - disagree (2)	0.99	[0.85 - 1.16]	1.05	[0.84 - 1.31]	0.95	[0.82 - 1.11]
Policy on air pollution aimed to improve wellbeing- agree (baseline)	1	[0 - 0]	1	[0 - 0]	1	[0 - 0]
Policy on air pollution aimed to improve wellbeing neutral (1)	1.30	[1.13 - 1.50]	1.50	[1.22 - 1.85]	1.47	[1.27 - 1.70]
Policy on air pollution aimed to improve wellbeing disagree (2)	0.91	[0.76 - 1.08]	1.01	[0.79 - 1.30]	0.91	[0.77 - 1.08]
Severe air annoyance - yes	0.99	[0.81 - 1.23]	0.97	[0.72 - 1.31]	0.86	[0.70 - 1.06]
Severe traffic - yes	0.93	[0.75 - 1.17]	0.90	[0.65 - 1.24]	0.95	[0.76 - 1.19]

### Variables associated with DK for noise

The factors associated with the DK for noise in the multiple logistic regression analysis are shown in Table 4. Similar to air pollution, social-demographic variables such as age, gender and education played a role in determining the DK for WTP. In addition, household income, awareness of health effects associated with noise and respondent’s view on the government’s role to reduce pollutants had a significant association in all WTP questions. However, there were more variables associated with the DK for the WTP general questions, e.g. environmental concerns, severe noise concerns, sensitivity towards noise and severe noise annoyance compared to the specific effect and the scenario WTP. Respondents answering the WTP general were more likely to cast a DK response when they were concerned about the environment and noise as pollutant, not sensitive towards noise, and were neutral about the role of the government to reduce noise. The Spanish and the Finnish respondents were more likely to provide DK for the WTP scenario question. Similar to air pollution, the degree of annoyance due to noise and the severe freight traffic nearby dwelling had an odds ratio of below 1, but did not reach statistical significance in most cases. FP is not significant for noise WTP.

**Table 4:** Odd ratios and confidence interval of variables associated with DK for noise in multiple logistic regression analysis

	**Noise**					
	**DK WTP general**		**DK WTP for severe annoyance**		**DK WTP scenario**	
	**O.R.**	**95% C.I. for O.R.**	**O.R.**	**95% C.I. for O.R.**	**O.R.**	**95% C.I. for O.R.**
Age group						
18-24 (baseline)	1	[0–0]	1	[0–0]	1	[0–0]
25-34 (1)	1.23	[1.00 - 1.52]	1.06	[0.79 - 1.43]	1.59	[1.29 - 1.96]
35-44 (2)	1.87	[1.53 - 2.29]	1.71	[1.28 - 2.28]	2.08	[1.69 - 2.55]
45-54 (3)	2.03	[1.65 - 2.50]	1.90	[1.41 - 2.55]	2.36	[1.92 - 2.91]
55-64 (4)	2.05	[1.65 - 2.54]	2.05	[1.51 - 2.78]	2.19	[1.76 - 2.73]
Gender male (baseline)	1	[0–0]	1	[0–0]	1	[0–0]
Gender female (1)	1.50	[1.33 - 1.69]	1.71	[1.44 - 2.04]	1.64	[1.45 - 1.86]
Education per 10 years	0.82	[0.72 - 0.92]	0.72	[0.60 - 0.86]	0.73	[0.64 - 0.82]
Country NL (baseline)	1	[0–0]	1	[0–0]	1	[0–0]
Country UK (1)	0.99	[0.81 - 1.21]	0.80	[0.61 - 1.06]	0.86	[0.70 - 1.05]
Country DE (2)	1.00	[0.82 - 1.22]	1.07	[0.80 - 1.42]	0.99	[0.81 - 1.21]
Country ES (3)	1.35	[1.09 - 1.68]	1.20	[0.88 - 1.63]	1.34	[1.07 - 1.66]
Country FI (4)	1.12	[0.92 - 1.36]	1.14	[0.86 - 1.51]	1.30	[1.06 - 1.59]
Financial position - not a problem at all (baseline)	1	[0–0]	1	[0–0]	1	[0–0]
FP – not a problem, but have to be careful (1)	0.96	[0.82 - 1.12]	1.10	[0.88 - 1.38]	0.87	[0.74 - 1.02]
FP – with a slight difficulty (2)	1.04	[0.87 - 1.23]	1.14	[0.89 - 1.46]	0.92	[0.78 - 1.10]
FP – with a large difficulty (3)	1.02	[0.81 - 1.28]	1.24	[0.89 - 1.73]	0.87	[0.69 - 1.10]
Household net income: €1000 or less (baseline)	1	[0–0]	1	[0–0]	1	[0–0]
Hh income €1001 to €1500 (1)	0.82	[0.68 - 0.99]	0.68	[0.52 - 0.89]	0.72	[0.60 - 0.87]
Hh income €1501 to €2500 (2)	0.73	[0.60 - 0.89]	0.73	[0.55 - 0.96]	0.68	[0.56 - 0.83]
Hh income €2001 to €3000 (3)	0.68	[0.57 - 0.83]	0.62	[0.47 - 0.82]	0.62	[0.51 - 0.76]
Hh income > €3001 (4)	0.70	[0.58 - 0.85]	0.63	[0.48 - 0.84]	0.57	[0.47 - 0.69]
General health score per 25	1.05	[0.97 - 1.14]	1.03	[0.92 - 1.15]	1.01	[0.94 - 1.10]
Awareness of health effects associated with noise - aware	0.88	[0.77 – 1.00]	0.76	[0.63 - 0.91]	0.84	[0.74 - 0.96]
Environmental concern: Low (baseline)	1	[0–0]	1	[0–0]	1	[0–0]
Environmental concern: Medium (1)	1.31	[1.12 - 1.53]	1.20	[0.96 - 1.50]	1.15	[0.98 - 1.35]
Environmental concern: High (2)	1.00	[0.84 - 1.18]	0.85	[0.67 - 1.09]	0.79	[0.67 - 0.94]
Severe noise concerns – yes (1)	1.21	[1.00 - 1.45]	1.11	[0.85 - 1.44]	1.09	[0.90 - 1.31]
Sensitive to noise – yes	0.83	[0.72 - 0.97]	0.97	[0.79 - 1.20]	0.98	[0.84 - 1.14]
Difficulty to relax in a place with noise - yes	1.06	[0.91 - 1.23]	0.96	[0.78 - 1.18]	0.90	[0.77 - 1.04]
Government doing their best to reduce noise - agree (baseline)	1	[0–0]	1	[0–0]	1	[0–0]
Government doing their best to reduce noise - neutral (1)	1.46	[1.24 - 1.73]	1.26	[0.99 - 1.60]	1.38	[1.16 - 1.64]
Government doing their best to reduce noise - disagree (2)	1.06	[0.90 - 1.24]	0.93	[0.74 - 1.16]	1.06	[0.91 - 1.25]
Policy on noise aimed to improve wellbeing- agree (baseline)	1	[0–0]	1	[0–0]	1	[0–0]
Policy on noise aimed to improve wellbeing neutral (1)	1.14	[0.98 - 1.32]	1.09	[0.89 - 1.35]	1.22	[1.05 - 1.42]
Policy on noise aimed to improve wellbeing disagree (2)	1.02	[0.86 - 1.21]	0.79	[0.62 – 1.00]	0.92	[0.77 - 1.09]
Severe noise annoyance - yes	0.81	[0.65 - 1.01]	0.84	[0.61 - 1.16]	0.84	[0.68 - 1.05]
Severe traffic - yes	0.92	[0.73 - 1.16]	0.87	[0.63 - 1.21]	0.73	[0.58 - 0.93]

### Influence of DK response on the distribution of WTP

**Figure 1:**
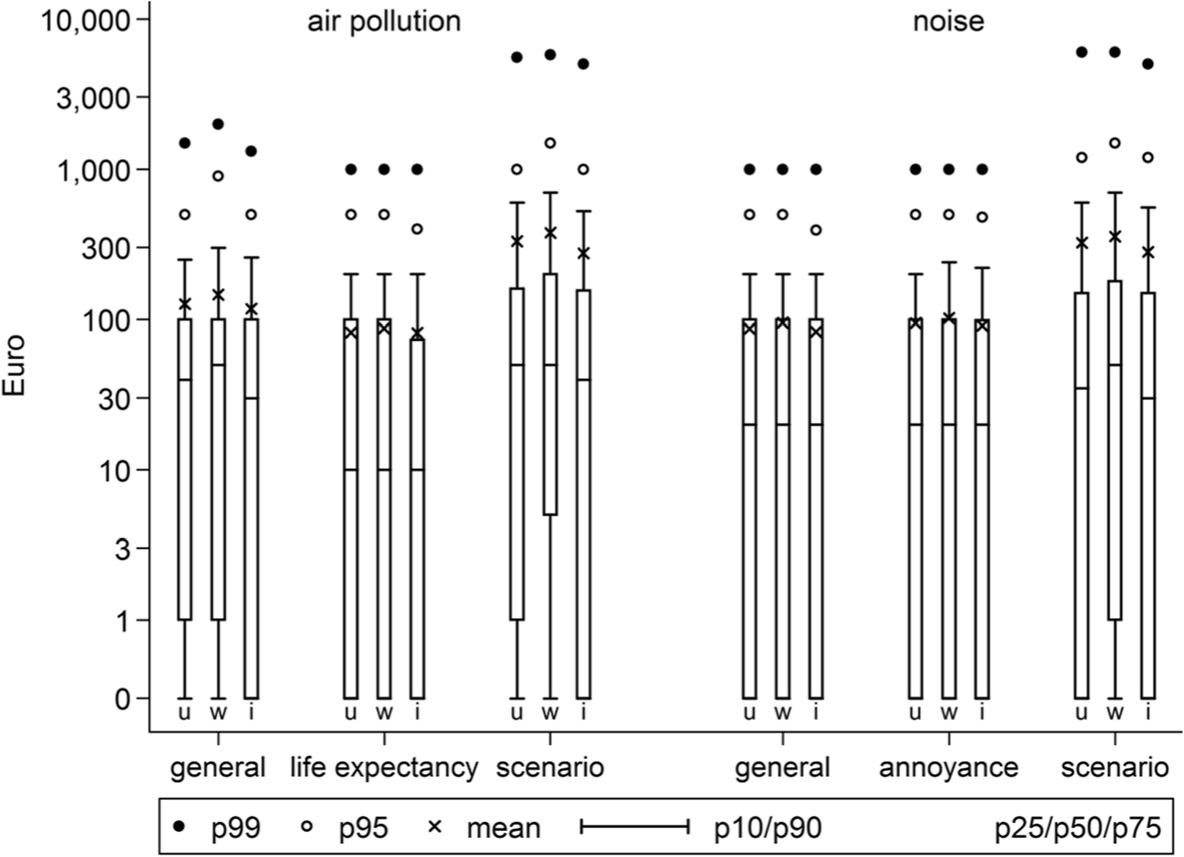
WTP estimates for general effects, a specific effect and a scenario of combined health effects related to air pollution and noise (u = un-weighted or unadjusted; w = weighted; and i = imputed).

To explore the possible influence of DK on the distribution of WTP values, we compared un-weighted, weighted and imputed WTP values. Figure 1 presents the distributions as boxplots. For general and specific effects, and a scenario of combined effects; the average WTP estimates of the un-weighted WTP did not differ substantially from the weighted and imputed WTP values. Figure 1 shows that by weighting and imputation, the WTP estimates for air pollution-related general effects and life expectancy, and for noise-related general effects and annoyance, remained more or less similar in the lower, middle and upper quartiles. The largest difference in estimates occurred by imputation due to an increase in the fraction with zero WTP.

## Discussion

We investigated in five different European countries the willingness-to-pay (WTP) to avoid the health effects associated with traffic-related air pollution and noise simultaneously using a single instrument: an open-ended web-based questionnaire. Specific research questions addressed the proportion of protest votes and don’t know answers, the determinants thereof and the effect of don’t know answers on the distribution of WTP. With approximately 5,000 respondents for air pollution and noise parts of questionnaire each, our study is larger than most other monetisation studies of environmental health effects. To our knowledge, there are no other multi-country studies of this scale reported in the open literature that simultaneously investigated the monetary value of health effects of both air pollution and noise through WTP with a single instrument and approach.

### Main findings

The proportion of PV was about 10%, with limited variation between countries. England and Finland had relatively lower proportion of PV compared to the other countries. About a third of PV were because respondents thought that costs should be included in transportation prices, i.e. the polluter should pay, with approximately another third because respondent thought that the government should pay costs to reduce levels. About one fifth of PV’s were principally against putting an amount of money on health. This indicates that the WTP concept is not fully aligned with judicial and ethical principles such as “the polluter pays”. Questions on willingness-to-accept (WTA), a concept that does not conflict with “the polluter pays” principle, had about 2% PV (data not shown). Between 47% and 56% of the respondents opted for the “don’t know (DK)” response and did not provide a value for the WTP questions. Differences in DK between the three vignettes were modest with 4–8% higher DK proportions for the vignettes with quantitative information. While the percentage DK answers may appear to be high, it is in line with response to questions about their future health. For example, 35% of the respondents answered with “don’t know” on the statement: “How true or false is the following statement for you: I expect my health to get worse?” It appears to be difficult to answer questions regarding one’s future (health) situation, let alone putting an economic value on future health situations. This in line with the Construal-level Theory of Psychological Distance in social psychology that suggests that due to the psychological distance, temporal distal situations are more difficult to assess than more proximal situations [36-38]. We also looked at the DK proportion in questions about WTA (data not shown). Interestingly enough, the DK for WTA was even higher with 56% than for the similar WTP question (48%).

We explored determinants of PV and DK based on sets of variables known to affect WTP and risk perception and acceptability. Several factors significantly influenced respondents PV and DK responses. In addition to social-demographic, economic and health factors known to affect WTP, environmental concern, awareness of health effects, respondent’s ability to relax in polluted places, and their view on the government’s role to reduce pollution and on policy to improve wellbeing, also affected PV and DK responses.

With the higher proportion of DK questions than PV, we explored the potential effect of DK on the distribution of WTP values, using weighing and imputation approaches. The results showed slightly different results but did not indicated substantial changes in the estimates of mean or median WTP, with differences of about 15% between un-weighted, weighted and imputed estimates.

### Methodological considerations and comparison to other multi-country studies

We used an open-ended web-based questionnaire to assess the WTP. Alternative CV approaches may have yielded somewhat different results. The debate about the best form to assess WTP for the valuation of the environmental related health effects is on-going and the choice of format (e.g. open-ended questions, payment cards, or discreet choice experiments) depends on the context and the research objectives [39-41]. The open-ended/OE method is reported as stable over time (high test-retest rate) and is considered to be free of anchoring and range biases [9] compared to other methods such as the payment card or the dichotomous choice method [15,42]. Avoiding anchoring and range bias effects was important to our objective to simultaneously assess the WTP for a diversity of health effects of air pollution and noise that differ in degrees of severity to the individual, family, and population at large. Thus, the OE-approach was the method of choice in this study, given its favourable features in this respect.

Compared to other contingent valuation methods such as discrete choice experiment and payment card method, the OE questionnaire method is generally reported to yield lower (conservative) WTP values [31,43,44] and may have a better construct validity. A disadvantage of the OE question approach is that it does not provide a smooth distribution of WTP values, since respondents tend to report ‘rounded’ numbers of fives, tens or hundreds and not the intermediate values. This drawback, however, did not interfere with our objectives.

The definition of PV in this study was based on the combination of a zero WTP value, combined with respondents holding ‘protest beliefs’. Respondents who gave unrealistically high WTP values, possibly as a way of a protest vote, were excluded from further analyses through the use of a 1.5% cut-off of the highest values, in line with the literature [21]. These high excluded values were, however, not included in our PV definition, but may be the result of similar underlying protest beliefs against the WTP concept. Also, we cannot exclude that respondents with protest beliefs may have answered a don’t know/DK answer. Nonetheless, the proportion of about 10% PV in our study compare well with those of a similar multi-country study on air pollution, the NEEDS study, which reported an overall 11% PV [21].

The literature on DK-answers in contingent valuation WTP studies is relatively underdeveloped and only a handful of studies allow DK answers or combine DK with “Don’t want to”, i.e. protest votes. There are few examples for comparison with our DK proportions in similar CV studies. The NEEDS study did not provide the DK-option to their respondents, but did ask how confident respondents were about their WTP answer. Some 35% responded that they were not confident, or had a missing value on this question [21]. In the multi-country HEATCO study on noise annoyance 3% of respondents indicated “I don’t know/I don’t want to answer the WTP question” [22]. It was thus not possible to distinguish between PV and DK in HEATCO. The 3% value, however, is clearly below our PV and DK values.

There is little empirical evidence about determinants of PV and DK from similar CV studies on environmental health effects. In the absence of a clear conceptual framework the observed associations should be considered as exploratory in nature. Many determinants for PV and DK were quite similar also across both pollutants, e.g. age, gender, country, financial position, familiarity with effects, environmental concern, difficulty to relax in polluted places, opinion on government’s attempt to reduce pollutants. Particularly the observed associations with variables known from the social sciences risk perception literature for both PV and DK warrant further study.

The weighting and imputation exercise did not suggest substantial deviations in the distribution of WTP. We cannot exclude, however, that unobserved factors may have influenced the probability of a DK-response and were not picked up in the weighting and imputation. Therefore, these exercises could not fully capture the effects of DK on WTP. Moreover, in our study the questionnaire was a kind of ‘thought experiment’ where respondents had nothing to gain or lose by their answers. In other situations, e.g. with contested policy issues where actual policy may be based on WTP studies, this may be different and PV and DK answers may indeed substantially affect the outcome of the WTP study. PV and DK answers and their determinants therefore deserve further study in CV studies on environmental health effects.

While this paper focuses on willingness and ability to provide quantitative WTP estimates for traffic-related health effects of air pollution and noise, it allows (un-weighted) WTP values for individual single health effects to be compared to similar results from multi-country CV studies on air pollution or noise. The NEEDS study on air pollution reported WTP estimates for an average for 6 months life expectancy gain of €384 pp/year with a median of €144 pp/year. This estimate is clearly much higher than the WTP in our study for an average gain of 6 months life expectancy (€82 pp/year and a median of €10 pp/year). Apart from other differences between the studies, the higher estimates may be in the line with the literature that open-ended contingent valuation method generally yields lower WTP values. The multi-country HEATCO study on noise reported a mean WTP of €50 pp/year, half of the mean in our study (€100 pp/year), to avoid the severe noise annoyance at that current moment. The WTP median was €0 pp/year compared to our median of €20 pp/year. Again, direct comparison is difficult due to differences in methods (open-ended vs. payment method), differences in sampling: open vs. stratified on noise levels, and differences in payment vehicle may also have affected responses. The differences are not in line with the literature that open-ended contingent valuation method generally yields lower WTP values.

## Conclusions

The phenomena of protest votes and particularly of uncertainty/don’t know responses are understudied; empirical evidence of determinants is largely absent. With a proportion of about 50%, DK answers may be more relevant issue affecting WTP than PV’s, although exploratory analysis in this study did not show substantial effects on the WTP distribution. The DK results did not differ much between the three vignettes; providing more and more quantitative information about effects did not reduce the percentage of “don’t know” response. The likelihood of protest vote and “don’t know” response was influenced by social-demographic, economic and health factors, people’s awareness of effects, environmental concerns, and appreciation of environmental conditions and policies. The “don’t know” responses hardly affected the distribution of the WTP answers in this study. This may, however, be different in other studies with other contexts, e.g. in the case of controversial environmental policy issues. Therefore, we recommend that in future research more attention should be paid not only to protest beliefs but also to the difficulties people may experience in expressing a monetary value for future environmental health situations. Explicit treatment of this phenomenon and its determinants is needed, particularly where results are used in real-life policy debates.

## Abbreviations

CI: Confidence interval; COI: Cost-of-illnesses; CV: Contingent valuation; DK: Don’t knows; HEATCO: Developing harmonious European approaches for transport costing and project assessment; INTARESE: Integrated assessment of health risks from environmental stressors in Europe; NEEDS: New energy externalities developments for sustainability; OE: Open-ended; OR: Odds ratio; PV: Protest vote; WTA: Willingness-to-accept; WTP: Willingness-to-pay.

## Competing interests

The authors declare that they have no competing interests.

## Authors’ contributions

All authors contributed to the issue framing, design and execution of the international survey, and the appraisal of the relevant outcomes. All authors read and approved the final manuscript.

## Supplementary Material

Additional file 1 The supplemental information file includes information provided to respondents prior to the WTP questions and WTP questions related to air pollution and noise used in the survey as discussed in the Method section. Click here for file

Additional file 2: Table S1 This table includes the determinants of PV for road-traffic air pollution and noise WTP. Click here for file
