# Supplementary material for: Multi-country willingness to pay study on road-traffic environmental health effects: are people willing and able to provide a number?
Source: Environ Health. 2014 May 9;13:35. doi: 10.1186/1476-069X-13-35 (PMC4030277; doi:10.1186/1476-069X-13-35)
Supplement: Additional file 1 — The supplemental information file includes information provided to respondents prior to the WTP questions and WTP questions related to air pollution and noise used in the survey as discussed in the Method section. [file 1476-069X-13-35-S1.docx]

**Appendix 1**

**Information provided to respondents prior to the WTP questions**

| **Information on effects of air pollution** | **Information on effects of noise** |
| --- | --- |
| Air pollution from road traffic has a number of negative health effects, according to many studies and authorities such as the World Health Organization (WHO). Road traffic air pollutants can cause childhood asthma, respiratory and cardiovascular diseases. All of these effects can shorten your life expectancy (the number of years you are expected to live). | Road traffic noise has a number of negative health and wellbeing effects according to many studies and authorities such as the World Health Organization (WHO). |
| Cardiovascular diseases are the disorder of the heart and blood vessels. They can limit your daily activity and worsen your quality of life. Patients with a respiratory disease are often limited in daily physical functioning, feel tired and exhausted by the attacks of coughing, shortness of breath and difficulty in breathing. | For heart disease, the most important risk of noise is a heart attack (myocardial infarction). Patients who have suffered a heart attack continue to rely on lifelong medical care. |
| Life expectancy is the expected number of years of life remaining at a given age. If you were born today, your life expectancy would be about 80 years. Women live on average 6 years longer than men. | The effects of noise on wellbeing include annoyance and sleep disturbance. Sleep disturbance is having trouble falling asleep, waking up irregularly in the middle of the night and/or waking up earlier than usual.  Annoyance is the feeling of disgust, anger, discomfort, dissatisfaction or upset that occurs when a person’s thoughts, feelings or activities, are negatively affected by the environment. |
| Cardiovascular and respiratory related diseases and life expectancy are not only related to air pollution, but they also depend on personal factors such as age, gender, weight, lifestyle and genetic makeup. Therefore, the elimination of air pollution does not necessarily lead to the elimination of the given diseases. | Research has also shown that noise can cause poorer reading performance in children at school. |

**Questions regarding specific effects and a scenario of combined effects**

**1 For WTP questions related to air pollution**

Some measures can be taken to reduce the current level of air pollution by road traffic. These measures require funding and the government will only cover a part of it. The rest of the expenses must therefore be brought up by the society in general. We are interested to know whether you are willing to pay for cleaner air due to road traffic to avoid the negative health effects.

The amounts of money you indicate to pay in the following questions come from your own budget. This means a cutback in your daily consumption of goods and services and/or savings.

**1.1 For general question on road traffic-related air pollution:**

Based on your indicated monthly net income, the net income of your household per year is: <referred to respondents indicated household income>

What is the maximum amount of money you personally are willing to pay annually for the rest of your life to avoid the effects of air pollution from road traffic in your living environment?

The maximum amount of money I am willing to pay is..... € or £ | per year

**1.2 For specific health effect on road traffic-related air pollution:**

A decrease with 50% of the air pollution emissions by 2030 will increase the life expectancy with 6 months.

What is the maximum amount of money you personally are willing to pay annually for the rest of your life to gain 6 months in life expectancy?

The maximum amount of money I am willing to pay is..... € or £ | per year

**1.3 For combined health effects on road traffic-related air pollution:**

In this scenario, a total reduction of 50% by 2030 in the emission of air pollutants from road traffic can be gradually achieved. After 2030, the emission of air pollutants will be maintained at this lower level.

The scenario will lead to:

• An increase of 6 months in life expectancy

• A reduction in your risk of hospital admission for cardiovascular diseases by 1 to 2 per year per 10,000 people

• A reduction in your risk of hospital admission for respiratory diseases by 1 to 2 per year per 10,000 people

• A reduction in the risk for doctor-diagnosed asthma in young children by 34 per year per 10,000 children

The benefits occur simultaneously and should be seen as a whole.

What is the maximum amount of money you personally are willing to pay annually for the rest of your life to benefit from this scenario?

The maximum amount of money I am willing to pay is..... € or £ | per year

**2 For WTP questions related to noise**

Some measures can be taken to reduce the current of level road traffic noise. These measures require funding and the government will only cover a part of it. The rest of the expenses must therefore be brought up by the society in general. We are interested to know whether you are willing to pay for less road traffic noise to avoid the negative health effects. The amounts of money you indicate to pay in the following questions come from your own budget. This means a cutback in your daily consumption of goods and services and/or savings.

**2.1 For general question on road traffic-related noise:**

Based on your indicated monthly net income, the net income of your household per year is: <referred to respondents indicated household income>

What is the maximum amount of money you personally are willing to pay annually for the rest of your life to avoid the effects of road traffic noise in your living environment?

The maximum amount of money I am willing to pay is..... € or £ | per year

**2.2** **For specific health effect on road traffic-related noise:**

Consider that a medium-size road is going to be built at a 75 meter distance from your home. Cars, motors, scooters, trucks and busses are allowed on these roads.

After this road is built, the annual noise level at your home will increase from 50 dB to 65 dB. This increase in noise level can affect for your health and wellbeing.

The noise will increase your risk of being severely annoyed due to traffic noise by 13% (13 per 100 people).

What is the maximum amount of money you personally are willing to pay annually for the rest of your life to avoid an additional risk of 13% to become severely annoyed due to traffic noise?

The maximum amount of money I am willing to pay is..... € or £ | per year

**2.3 For combined health effects on road traffic-related noise:**

Consider that a medium-size road is going to be built at a 75 meter distance from your home. Cars, motors, scooters, trucks and busses are allowed on these roads.

After this road is built, the annual noise level at your home will increase from 50 dB to 65 dB.

The scenario will lead to:

• An increase of your risk with 13% to become severely annoyed due to road traffic

• An increase of your risk with 6% to have severe sleep disturbance at night due to road traffic noise

• An increase of your risk for a heart attack by 1 per year per 10,000 people

• An increase in the risk of poorer reading performance by children during their primary education by 100 per 10,000 children

The effects occur simultaneously and should be seen as a whole.

What is the maximum amount of money you personally are willing to pay annually for the rest of your life to avoid the negative effects of this scenario?

The maximum amount of money I am willing to pay is..... € or £ | per year
