# Supplementary material for: Multi-country willingness to pay study on road-traffic environmental health effects: are people willing and able to provide a number?
Source: Environ Health. 2014 May 9;13:35. doi: 10.1186/1476-069X-13-35 (PMC4030277; doi:10.1186/1476-069X-13-35)
Supplement: Additional file 2: Table S1 — This table includes the determinants of PV for road-traffic air pollution and noise WTP. [file 1476-069X-13-35-S2.docx]

**Appendix 2**

**Table 3: Determinants of PV for road-traffic air pollution and noise WTP**

|  | **Air pollution PV** | | **Noise PV** | |
| --- | --- | --- | --- | --- |
|  | **O.R.** | **95% C.I. for O.R.** | **O.R.** | **95% C.I. for O.R.** |
| Age group |  |  |  |  |
| 18-24 (baseline) | 1 | [0 - 0] | 1 | [0 - 0] |
| 25-34 (1) | 1.26 | [0.84 - 1.90] | 2.54 | [1.71 - 3.77] |
| 35-44 (2) | 1.75 | [1.19 - 2.58] | 2.39 | [1.62 - 3.55] |
| 45-54 (3) | 2.25 | [1.53 - 3.32] | 2.86 | [1.93 - 4.23] |
| 55-64 (4) | 1.71 | [1.13 - 2.61] | 3.51 | [2.36 - 5.22] |
| Gender female (1) | 0.81 | [0.66 - 1.00] | 0.84 | [0.70 - 1.01] |
| Education per 10 years | 1.25 | [1.05 - 1.49] | 0.92 | [0.77 - 1.11] |
| Country NL (baseline) | 1 | [0 - 0] | 1 | [0 - 0] |
| Country UK (1) | 0.65 | [0.47 - 0.91] | 0.78 | [0.58 - 1.04] |
| Country DE (2) | 1.09 | [0.80 - 1.47] | 0.97 | [0.74 - 1.29] |
| Country ES (3) | 1.08 | [0.77 - 1.52] | 0.93 | [0.67 - 1.28] |
| Country FI (4) | 0.44 | [0.30 - 0.64] | 0.61 | [0.44 - 0.83] |
| Financial position - not a problem at all (baseline) | 1 | [0 - 0] | 1 | [0 - 0] |
| FP – not a problem. but have to be careful (1) | 1.14 | [0.85 - 1.51] | 1.00 | [0.78 - 1.28] |
| FP – with a slight difficulty (2) | 1.34 | [0.98 - 1.81] | 1.23 | [0.95 - 1.60] |
| FP – with a large difficulty (3) | 2.31 | [1.64 - 3.26] | 1.80 | [1.32 - 2.45] |
| Household net income: €1000 or less (baseline) | 1 | [0 - 0] | 1 | [0 - 0] |
| Hh income €1001 to €1500 (1) | 0.91 | [0.67 - 1.24] | 0.98 | [0.74 - 1.28] |
| Hh income €1501 to €2500 (2) | 1.19 | [0.88 - 1.61] | 0.92 | [0.69 - 1.23] |
| Hh income €2001 to €3000 (3) | 0.70 | [0.50 - 0.97] | 0.97 | [0.73 - 1.29] |
| Hh income > €3001 (4) | 0.84 | [0.60 - 1.18] | 0.74 | [0.55 - 1.01] |
| General health score per 25 | 1.12 | [0.91 - 1.37] | 1.20 | [1.07 - 1.35] |
| Awareness of health effects associated with air pollution/noise - aware | 0.94 | [0.76 - 1.16] | 0.92 | [0.75 - 1.12] |
| Environmental concern: Low (baseline) | 1 | [0 - 0] | 1 | [0 - 0] |
| Environmental concern: Medium (1) | 0.48 | [0.37 - 0.61] | 0.66 | [0.53 - 0.82] |
| Environmental concern: High (2) | 0.43 | [0.32 - 0.57] | 0.45 | [0.35 - 0.58] |
| Severe air pollution/noise concerns –yes (1) | 1.02 | [0.75 - 1.39] | 1.12 | [0.85 - 1.48] |
| Sensitive to air/noise pollution – yes | 0.96 | [0.72 - 1.27] | 0.98 | [0.78 - 1.23] |
| Difficulty to relax in a place with air pollution/noise - yes | 0.58 | [0.46 - 0.73] | 0.84 | [0.68 - 1.05] |
| Government doing their best to reduce air pollution/noise - agree (baseline) | 1 | [0 - 0] | 1 | [0 - 0] |
| Government doing their best to reduce air pollution/noise - neutral (1) | 1.08 | [0.79 - 1.48] | 1.17 | [0.88 - 1.56] |
| Government doing their best to reduce air pollution/noise - disagree (2) | 2.18 | [1.65 - 2.89] | 2.04 | [1.58 - 2.63] |
| Policy on air pollution/noise aimed to improve wellbeing- agree (baseline) | 1 | [0 - 0] | 1 | [0 - 0] |
| Policy on air pollution/noise aimed to improve wellbeing neutral (1) | 0.92 | [0.72 - 1.18] | 0.72 | [0.57 - 0.91] |
| Policy on air pollution/noise aimed to improve wellbeing disagree (2) | 0.87 | [0.65 - 1.17] | 1.01 | [0.79 - 1.29] |
| Severe air/noise annoyance- yes | 0.90 | [0.62 - 1.30] | 0.89 | [0.64 - 1.23] |
| Severe traffic- yes | 1.03 | [0.71 - 1.48] | 1.50 | [1.10 - 2.05] |

Table 3 shows the factors associated with PV in the multiple logistic regression analysis. Age (except for age group 25-34 years old answering air pollution questions), gender, countries (UK and Finland), large difficulties with financial position, higher income groups, environmental concern, respondent’s perception that government was not doing their best to reduce pollutants, were significantly associated with WTP PV for air pollution and noise.

In addition, specifically, respondents with lower education, middle household income group, and those who indicated that they had no difficulty to relax in polluted places, were the least likely to carry a PV to air pollution WTP question.

Specifically for noise WTP PV, respondents in the highest household income group and those feeling less healthy, agreeing that government was doing their best to reduce noise, were neutral regarding the noise policy aimed to improve the wellbeing, and perceiving less severe freight traffic, were the least likely to carry a PV.
